# Placenta Accreta Spectrum Prenatal Diagnosis Performance: Are Ultrasound False-positive Results Acceptable in Limited-resources Settings?

**DOI:** 10.1055/s-0042-1751061

**Published:** 2022-09-06

**Authors:** Albaro José Nieto-Calvache, Juan Pablo Benavides-Calvache, Alejandra Hidalgo, Natalia Padilla, Jaime López-Tenorio, Alejandro Victoria, Martin Rengifo, Mauricio Mejía, Lina María Vergara-Galliadi, Stiven Ernesto Sinisterra-Díaz, Juliana Maya, María Andrea Zambrano, Juan Manuel Burgos-Luna

**Affiliations:** 1 Clínica de Espectro de Acretismo Placentario, Fundación Valle del Lili, Cali, Colombia; 2 Centro de Investigaciones Clínicas, Fundación Valle del Lili, Cali, Colombia; 3 Departamento de Radiología, Fundación Valle del Lili, Cali, Colombia; 4 Facultad de Ciencias de la Salud, Programa de Medicina, Universidad Icesi, Cali, Colombia; 5 Facultad de Ciencias de la Salud, Programa de Ginecología y Obstetricia, Universidad Icesi, Cali, Colombia

**Keywords:** placenta accreta, ultrasonography, false positive, prenatal ultrasonic diagnosis, operative surgical procedure, placenta acreta, ultrassonografia, falso positivo, diagnóstico ultrassônico pré-natal, procedimento cirúrgico operatório

## Abstract

**Objective** The immediate referral of patients with risk factors for placenta accreta spectrum (PAS) to specialized centers is recommended, thus favoring an early diagnosis and an interdisciplinary management. However, diagnostic errors are frequent, even in referral centers (RCs). We sought to evaluate the performance of the prenatal diagnosis for PAS in a Latin American hospital.

**Methods** A retrospective descriptive study including patients referred due to the suspicion of PAS was conducted. Data from the prenatal imaging studies were compared with the final diagnoses (intraoperative and/or histological).

**Results** A total of 162 patients were included in the present study. The median gestational age at the time of the first PAS suspicious ultrasound was 29 weeks, but patients arrived at the PAS RC at 34 weeks. The frequency of false-positive results at referring hospitals was 68.5%. Sixty-nine patients underwent surgery based on the suspicion of PAS at 35 weeks, and there was a 28.9% false-positive rate at the RC. In 93 patients, the diagnosis of PAS was ruled out at the RC, with a 2.1% false-negative frequency.

**Conclusion** The prenatal diagnosis of PAS is better at the RC. However, even in these centers, false-positive results are common; therefore, the intraoperative confirmation of the diagnosis of PAS is essential.

## Resumo

**Objetivo** Recomenda-se o encaminhamento imediato de pacientes com fatores de risco para espectro placentário acreta (PAS, na sigla em inglês) para centros especializados, favorecendo assim o diagnóstico precoce e o manejo interdisciplinar. No entanto, erros diagnósticos são frequentes, mesmo em centros de referência (CRs). Buscou-se avaliar o desempenho do diagnóstico pré-natal para PAS em um hospital latino-americano.

**Métodos** Um estudo descritivo retrospectivo incluindo pacientes encaminhados por suspeita de SAP foi realizado. Os dados dos exames de imagem do pré-natal foram comparados com os diagnósticos finais (intraoperatórios e/ou histológicos).

**Resultados** Foram incluídos 162 pacientes no presente estudo. A idade gestacional mediana no momento da primeira ultrassonografia suspeita de PAS foi de 29 semanas, mas as pacientes chegaram ao CR de PAS com 34 semanas. A frequência de resultados falso-positivos nos hospitais de referência foi de 68,5%. Sessenta e nove pacientes foram operadas com base na suspeita de PAS com 35 semanas e houve 28,9% de falso-positivos no CR. Em 93 pacientes, o diagnóstico de PAS foi descartado no CR, com frequência de falso-negativos de 2,1%.

**Conclusão** O diagnóstico pré-natal de PAS é melhor no CR. Entretanto, mesmo nestes centros, resultados falso-positivos são comuns; portanto, a confirmação intraoperatória do diagnóstico de SAP é essencial.

## Introduction

Placenta accreta spectrum (PAS) can lead to severe complications among patients and can cause maternal death. 1 It is recommended that patients with risk factors for PAS receive timely transfers to referral centers (RCs), 2 facilitating additional diagnostic evaluations (ultrasonography and magnetic resonance imaging [MRI]) that confirm or rule out a PAS diagnosis, as well as the planning of the surgery with the participation of interdisciplinary groups.

Achieving healthcare team training in the management of PAS cases is difficult, and it takes several years of work and a large number of patients seen for the teams to consider their “training curve” fulfilled. 3 In low- and middle-income countries (LMICs), few hospitals have significant experience in the diagnosis and management of PAS, 4 and it has been reported that in approximately one-third of cases a prenatal diagnosis is not possible. 5 6 7 Therefore, it is common for patients with PAS to arrive at experienced centers late in the course of the disease or to be cared for at hospitals without the recommended resources. 8

We aimed to evaluate the accuracies of the prenatal diagnosis of PAS in a Latin American hospital, and we evaluated the time elapsed between the suspected diagnosis at the initial care hospitals and the specialized evaluation, as well as the number of diagnostic procedures used and their correlation with the final diagnosis.

## Methods

A retrospective descriptive study including patients referred to the Fundación Valle de Lili University Hospital (FVL), Cali, Colombia, due to suspected PAS between December 2016 and May 2021 was conducted. The FVL is a PAS referral center (PAS-RC). Data were obtained from the prenatal imaging studies performed since the suspected diagnosis of PAS was made until the pregnancy was finalized.

Aiming to describe the accuracy of the prenatal diagnosis in our PAS-RC, the patients were divided into two groups according to the treatment they received after the initial assessment, rather than to the actual presence of PAS (final diagnosis): Those treated by the PAS-RC as “PAS suspected by imaging” (Group 1) and those treated as “PAS not suspected by imaging” (Group 2). Upon arrival at our hospital, our PAS team tried to perform a diagnostic imaging study to verify the presence of PAS in the patients referred for this reason. We use ultrasound (US) as the first diagnostic method and we follow the European Working Group on Abnormally Invasive Placenta recommendations. 9 In some cases, it was also necessary to perform a placental magnetic resonance imaging (MRI); for its interpretation, we used the recommendations from Palacios Jaraquemada et al. 10

**Fig. 1:**
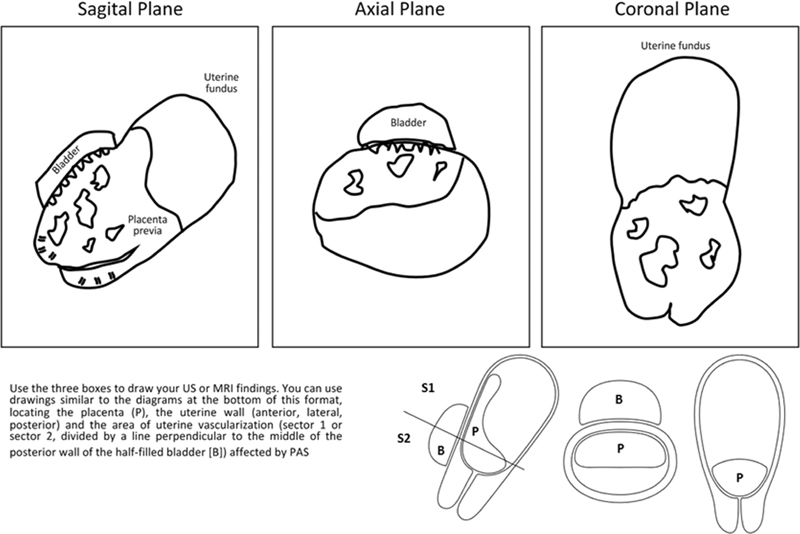
Imaging findings scheme (ultrasonography or magnetic resonance imaging) in suspected placenta accreta spectrum (PAS) cases. The scheme is used to improve communication between the prenatal diagnosis group (maternal-fetal specialist and/or radiologist) and the surgical group during the planning of the surgical procedure.

**Fig. 2:**
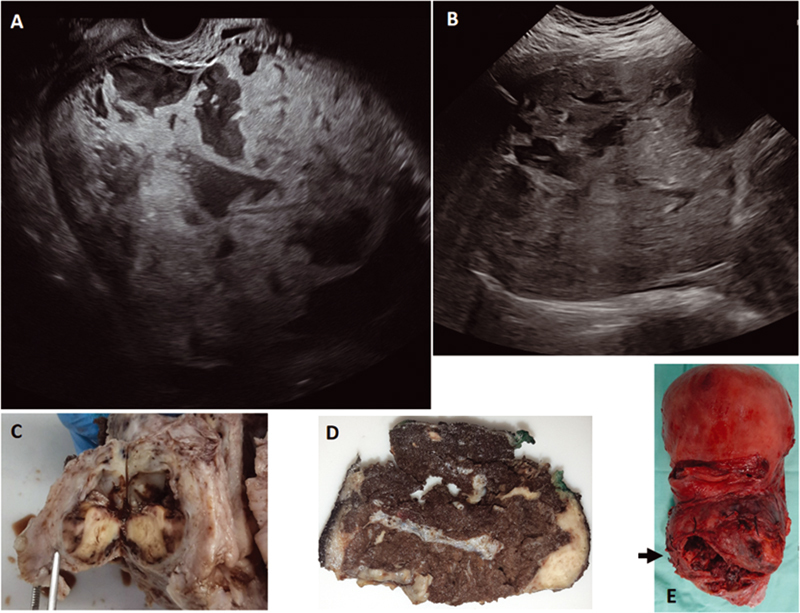
(A and B). Ultrasonography (US) images. (A): Sagittal plane section through the vagina, showing pathological lacunae with turbulent flow toward the cervix. (B): Axial plane section through the abdomen with loss of the uteroplacental interface and pathological placental lacunae; limited visualization due to abundant adipose tissue and scars on the abdominal wall (3 previous cesarean sections). (C to E). Uterus and placenta once resected. (C): Axial plane section at the level of the cervix, at the same level as the US image in A. Severe anatomical distortion can be seen, correlating with the presurgical drawing of the sagittal plane in Figure 1 . (D): Axial section of the lower uterine segment, at a level that corresponds to the US image in B and the level marked with an arrow in E. Severe thinning of the myometrium can be seen in the anterior part of the uterus, with areas of serosal loss, probably related to the surgical procedure. (E): Anterior face of the uterus showing correlation with the presurgical drawing of the coronal plane section on Figure 1 .

Additionally, our maternal fetal medicine specialist and radiologist presented the US and MRI findings visualized in a drawing of the uterus, of the placenta, and of the neighboring organs ( Fig. 1 ) to facilitate the integration of the US/MRI evaluation into a surgical plan with the entire PAS team (many of whose members do not fully understand US/MRI images) ( Fig. 2 ).

Inside each box, the specialist in prenatal diagnosis draws (guiding themselves by the drawings in the lower part of the format) their findings in the imaging study. This figure encompasses the ultrasonographic findings of a patient at 30 weeks of gestation with 3 previous cesarean sections, placenta previa and PAS, whose US and surgical findings are shown in Fig. 2 .

The frequency of false-positive and false-negative prenatal diagnoses at referring hospitals (RHs) and the RC were calculated by comparing the final postoperative diagnosis (applying the clinical International Federation of Gynecology and Obstetrics (FIGO) staging criteria and histopathology) 11 with the prenatal diagnosis issued at the RH and at the PAS-RC, respectively. Since the FIGO criteria were published in 2019, cases treated before that date were diagnosed as PAS if they were confirmed by postoperative histological analysis or if the clinical criteria described by Collins et al. were present during the cesarean section. 12

Descriptive statistics were used. Continuous variables were described by means of medians and interquartile ranges (IQRs), and categorical variables were described by relative and absolute frequencies. The present study was approved by the ethics committee and by the institutional review board (protocol No. 1023).

## Results

**Fig 3:**
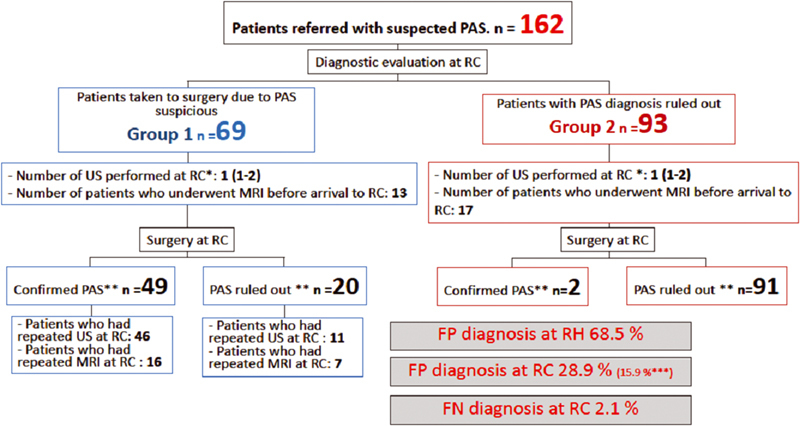
Flowchart of patients referred due to suspected placenta accreta spectrum (PAS) to a referral center (RC) and performance of prenatal diagnostic images. Abbreviations: FN, false-negative; FP, false-positive. MRI, magnetic resonance imaging; RH, referring hospital; US, ultrasonography. False-positive and FN values were calculated by comparing the presurgical diagnosis issued by the RH and the RC with the final postoperative diagnosis (applying clinical FIGO staging criteria and histopathology); * median (interquartile range); ** Confirmed or excluded PAS by intraoperative or histological findings. *** Percentage of FPs excluding patients for whom it was not possible to perform US in RC due to being admitted in an emergency situation.

A total of 162 patients were referred to the FVL after the RH had determined there was a prenatal suspicion of PAS, within a 64-month period ( Fig. 3 ). The median gestational age at the time of the first PAS suspicious US at the (RHs) was 29.1 (IQR: 24–33.5) weeks, and there were 2 suspicious US examinatiosns (IQR: 2–3). In 30 cases, MRI was also performed before the patients were transferred to the RC.

Patients arrived at the PAS-RC with an average gestational age of 34 (IQR: 29–36) weeks. Sixty-nine patients underwent surgery based on the suspicion of PAS (Group 1) at 35 (IQR: 34–37) weeks. Twelve of these patients (17.4% of the Group 1 cases) were admitted in an emergency condition (with vaginal bleeding or in labor), so it was not possible to perform any prenatal imaging in the PAS-RC before proceeding to perform an emergency surgery. The remaining 57 patients underwent US in the PAS-RC, and 23 of them also underwent an MRI. Forty-nine women in Group 1 had an intraoperative and/or a histological confirmation of PAS. In the remaining 20 patients, the final diagnosis ranged between placenta previa, uterine dehiscence, or normal placenta. In 93 patients, a diagnosis of PAS was ruled out (Group 2) by US (in 12 cases, an MRI was also performed), which was performed at the PAS-RC. The Group 2 pregnancies were finalized at week 36 (IQR: 34–38), and 2 patients had an intraoperative diagnosis for mild PAS (focal accreta), which corresponds to a false-negative rate of 2.1%. The false-positive rate at the RH was 68.5%. When comparing the diagnosis made by the PAS-RC with the final diagnosis, 28.9% of the diagnoses were false-positives. However, the false-positive rate would drop to 15.9% if 9 patients, for whom it was not possible to perform an US in the PAS-RC due to the late remission of the patients, were excluded. Table 1 shows how the frequency of false-positives decreased progressively between 2017 and 2021, decreasing from 45 to 14.3%.

**Table 1:** Ultrasound diagnostic performance for placenta accreta spectrum in a Latin American referral center

	2017	2018	2019	2020	2021 a	Total
Patients referred with suspected PAS	29	43	37	35	18	162
Patients managed with suspected PAS at the RC	20	15	16	11	7	69
Patients with PAS ruled out at the RC	9	28	21	24	11	93
False-positives, *n* (%)	9 (45)	4 (26.7)	4 (25)	2 (18.2)	1 (14.3)	20 (28.9) b
False-negatives, *n* (%)	0	1 (3.6)	1 (4.8)	0	0	2 (2.1)

Abbreviations: PAS, placenta accreta spectrum; RC, referral center.

a Between January and May 2021.

b This value decreases to 15.9% if only the 11 patients who underwent ultrasonography before surgery in the RH are considered (9 patients with prenatal suspicion of PAS and who finally did not have this diagnosis were admitted in emergency condition and it was not possible to perform ultrasound before surgery).

In 35 cases (23 in Group 1 and 12 in Group 2), an MRI was performed at the RC ( Fig. 3 ). Sixteen of these patients had a previous MRI performed at the RH (12 in Group 1 and 4 in Group 2), but the quality of the initial study did not allow RC radiologists to issue a concept about the diagnosis of PAS (elements such as empty bladder and axial planes not perpendicular to the posterior wall of the bladder were identified). In most cases, the MRI was repeated because the US assessment left doubts about the presence of parametrial or posterior uterine wall involvement.

## Discussion

In the present retrospective analysis of PAS patients referred to a Latin American hospital, we found that the accuracy of the prenatal diagnosis was higher in a PAS-RC compared with an initial care center. Ultrasonography is the most widely used diagnostic method for PAS; however, its performance depends (as well as the performance of the MRI) on the skills of the team performing and reading it and on the training and experience of the sonographer. 13 In our analysis, we found that the diagnosis of PAS established at basic levels of care (RHs) had a 68.5% false-positive rate ( Fig. 3 ). Although the main diagnostic strategy used was US, MRI was also used in some of these false-positive cases. The frequency of the false-positive results in our PAS-RC was 15.9%, with a decrease in frequency year after year as the interdisciplinary team improved its competencies ( Table 1 ).

The methodology used in the present study does not allow us to calculate the false-negative rate of USs performed at basic levels of care since not all of these patients were referred to our institution. False-negatives in the RH may be a major reason of missed PAS diagnosis, and studies with a larger population base are necessary to evaluate their incidence. However, 2.1% of the cases diagnosed in the PAS-RC were false negatives (2 cases of focal placenta accreta).

It may be alarming that 1 in 6 patients diagnosed with PAS using the prenatal images in a PAS-RC actually did not have it. However, after considering the obvious possibility of the patient dying if care is received from inexperienced healthcare groups, 14 we believe that this number of false positives may be acceptable in patients with potentially severe pathological conditions, especially when patients are at institutions where they are on the steep part of the “training curve” or in limited-resource settings.

The performances of both the entry level hospitals and the RCs should always be taken into consideration separately. A large number of false-positives in the screening setting (RH) is not a problem if all patients with PAS are detected and if there is an adequate regional referral service where the diagnosis can be confirmed or ruled out. 15 Even in the PAS-RC, it is better to have more false-positives than false-negatives, if there is a strategy to confirm the diagnosis during laparotomy, before implementing invasive interventions.

The year-by-year analysis of false-positives ( Table 1 ) shows that their frequency is decreasing, and this is probably related to an increase in the experience of the prenatal diagnosis teams (specialists in maternal-fetal medicine and radiologists). Although high specificity has been reported for experienced teams, 16 the false-positive frequency observed in our series is consistent with the publications from other centers, describing a specificity that varies between 0.68 and 0.8. 17 18 19

Our hospital has been a PAS-RC since 2011, but only since 2016 a PAS team that followed and applied quality policies and actively did research was officially formed. 20 Since that year, a record has been kept of all patients treated with a suspected diagnosis of PAS, including those whose diagnosis was ruled out after an US evaluation (previously, only those who were taken to surgery under the suspicion of PAS were followed-up). Although the methodology used in the present study does not allow us to evaluate the factors that explain the progressive improvement in the diagnostic efficacy of our PAS team, it is probably due to the stability of the sonographers (all cases were evaluated by the same two maternal-fetal medicine [MFM] specialists) and of the radiologists (all MRIs were read by the same two radiologists), better communication within the PAS team (including feedback from the surgical results and histological analysis, periodic review of the cases attended, including analysis of MRI and US images) and contact with other PAS teams (including performance audit by other PAS teams). 21

Because, usually, only confirmed cases of PAS are included in the analyses, many authors do not report the false-positive rate when using US as the diagnostic test for PAS. 22

It is essential to have an intraoperative confirmation of a prenatal PAS diagnosis before proceeding with additional interventions such as vascular procedures (endovascular balloons or pelvic vessel ligation) or even before starting the hysterectomy because there is a real possibility of having false-positives in the diagnosis of PAS. In our center, we have used “intraoperative staging,” 20 where the first step is to dissect the vesicouterine space and evaluate the anterior wall of the uterus to determine the presence or absence of PAS and evaluate its severity. Extensive laparotomies, fundic hysterotomies, and endovascular devices, which can have associated complications, 23 can be avoided if a PAS diagnosis is confirmed (or ruled out) intraoperatively.

Our observations also show that there is a 5-week period between a prenatal PAS suspicion at an RH and referral to a PAS-RC, with remission rates being high for emergency conditions (17.4% of the Group 1 cases).

Several expert groups recommend the immediate referral of a patient to expert centers when the patient presents with risk factors for PAS, even when there are inconclusive US findings suggestive of abnormal placentation. 8 However, referrals are frequently deferred, even in institutions without the necessary resources for PAS interdisciplinary care. 7 In the present series, patients contacted the PAS-RC around week 34, just 1 week before the recommended date to end the pregnancy in patients affected by PAS, 24 and this delay leaves less time for interdisciplinary planning.

Among the conditions that make a timely referral difficult in LMICs are administrative factors (type of health insurance) and geographic factors (large areas of the country without expert centers). 25 However, the most important factors are the lack of active search for PAS, the absence of a clearly defined treatment and referral protocols at the regional level, and the lack of recognition by an obstetrician of the importance of getting expert groups involved. 26

In addition to the fact that timely contact with PAS-RC is related to better clinical outcomes, 3 it is also related to a better use of health resources. The median number of US required to rule out or confirm PAS at the RC was one. Group 2 patients underwent previously 2 PAS-suggestive US in the RH, and sometimes (17 of these 93 patients) an MRI was also performed ( Fig. 3 ).

In 16 of the 35 patients who arrived at the RC with a previous MRI (performed at the RH), it was necessary to repeat the MRI because the initial study did not allow the RC radiologists to have adequate visualization of the structures and, thus, to have a high degree of certainty about a diagnosis of PAS (mainly on parametrial or posterior uterine wall involvement). Although it is not the scope of the present study, the observed results provide an opportunity to reduce the number of prenatal images required for the proper diagnosis of PAS if patients contact expert centers early.

The present study has limitations that must be considered when analyzing our results. Although data from the USs and MRIs that were performed prior to the admission of patients to the PAS-RC were included, this is a single-center study, and the validity of our observations may not be applicable to other populations. Our hospital is an RC for PAS; however, our interdisciplinary group is still in the steep segment of its “training curve,” and the accuracy of the prenatal diagnosis of PAS is likely to be different in other hospitals with either more or less experience than our hospital. We believe that reporting the difficulty in the prenatal diagnosis of PAS in an LMIC may be useful for other groups seeking to evaluate their performance on stablishing a prenatal diagnosis of PAS. Finally, the retrospective design of the present study allows for potential bias, and it is difficult with this type of design to fully ensure the use of uniform criteria for the diagnosis of PAS. However, our group applies the same US analysis and report protocol for all cases evaluated. 9

Evidently, there is a need to perform prospective multicenter studies that evaluate the diagnostic tests for PAS not only in PAS-RCs, but also at the regional levels.

## Conclusion

The accuracy of a prenatal diagnosis for PAS is higher in RCs. However, even in these centers, false-positive results are common, making the intraoperative confirmation of the diagnosis of PAS essential.
